# Supplementary material for: Proteome analysis of xylose metabolism in Rhodotorula toruloides during lipid production
Source: Biotechnol Biofuels. 2019 Jun 4;12:137. doi: 10.1186/s13068-019-1478-8 (PMC6547517; doi:10.1186/s13068-019-1478-8)
Supplement: Supplementary file 2 — Additional file 2: Fig. S1. GO term enrichment analysis and volcano plots of differentially expressed proteins in R. toruloides during either exponential growth phase or lipid production phase in medium containing either glucose (A) or xylose (B). For each GO term showing significant change (rank score of ≥ 5), the direction and significance of the relative changes in protein levels are shown, together with the total number of proteins within each GO term. Fig. S2. Venn diagram of differentially expressed proteins in R. toruloidesduring either exponential growth phase or lipid production phase in medium containing either glucose (yellow circle) or xylose (blue circle). Number in red or blue colour indicates up or downregulated proteins, respectively, in cells during lipid accumulation as compared to exponential phase. Fig. S3. Volcano plots of differentially expressed proteins in R. toruloides cultivated on either glucose or xylose during early exponential growth phase (A), late exponential growth phase (B), or lipid accumulation phase (C). Fig. S4. Venn diagram of differentially expressed proteins in R. toruloidescultivated on either glucose or xylose during early exponential growth phase (t1, yellow circle), late exponential growth phase (t2, brown circle), or lipid accumulation phase (t3, green cycle). Number in red or blue colour indicates up or downregulated proteins, respectively, in cells grown on xylose as compared to cells grown on glucose. [file 13068_2019_1478_MOESM2_ESM.pdf]

Fig. S1

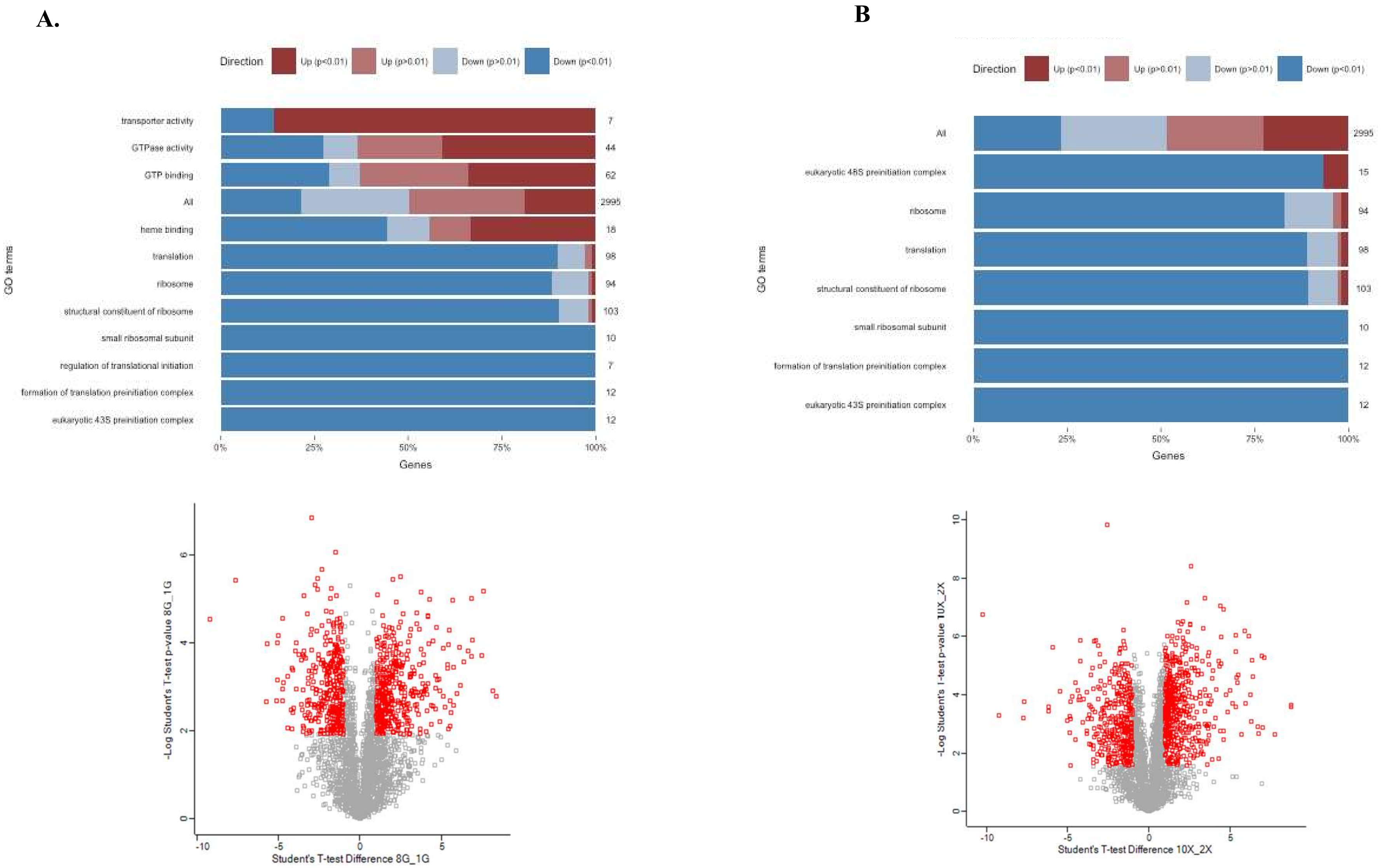

Fig. S1. GO term enrichment analysis and volcano plots of differentially expressed proteins in *R. toruloides* during either exponential growth phase or lipid production phase in medium containing either glucose (A) or xylose (B). For each GO term showing significant change (rank score of ≥ 5), the direction and significance of the relative changes in protein levels are shown, together with the total number of proteins within each GO term.

Fig. S2

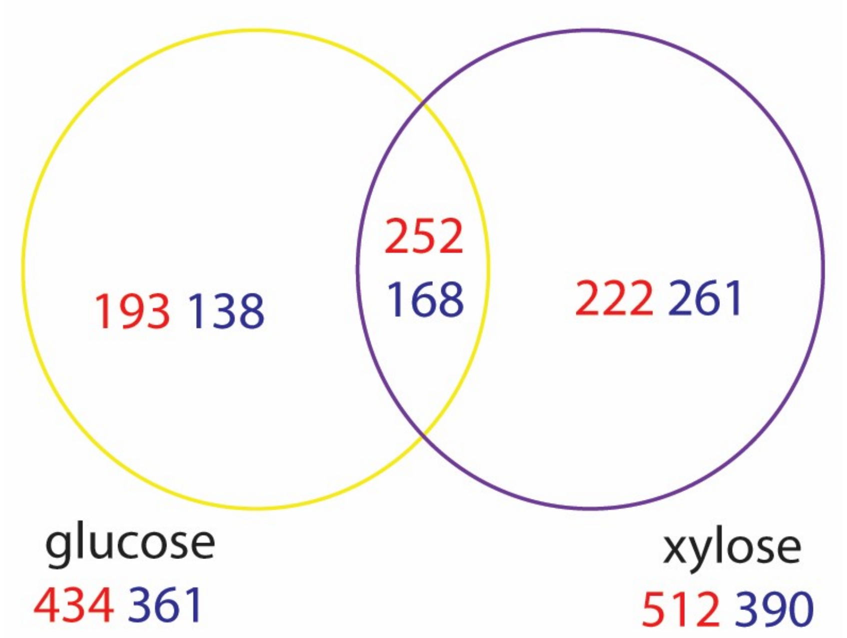

Fig. S2. Venn diagram of differentially expressed proteins in *R. toruloides* during either exponential growth phase or lipid production phase in medium containing either glucose (yellow circle) or xylose (blue circle). Number in red or blue colour indicates up or downregulated proteins, respectively, in cells during lipid accumulation as compared to exponential phase.

Fig. S3

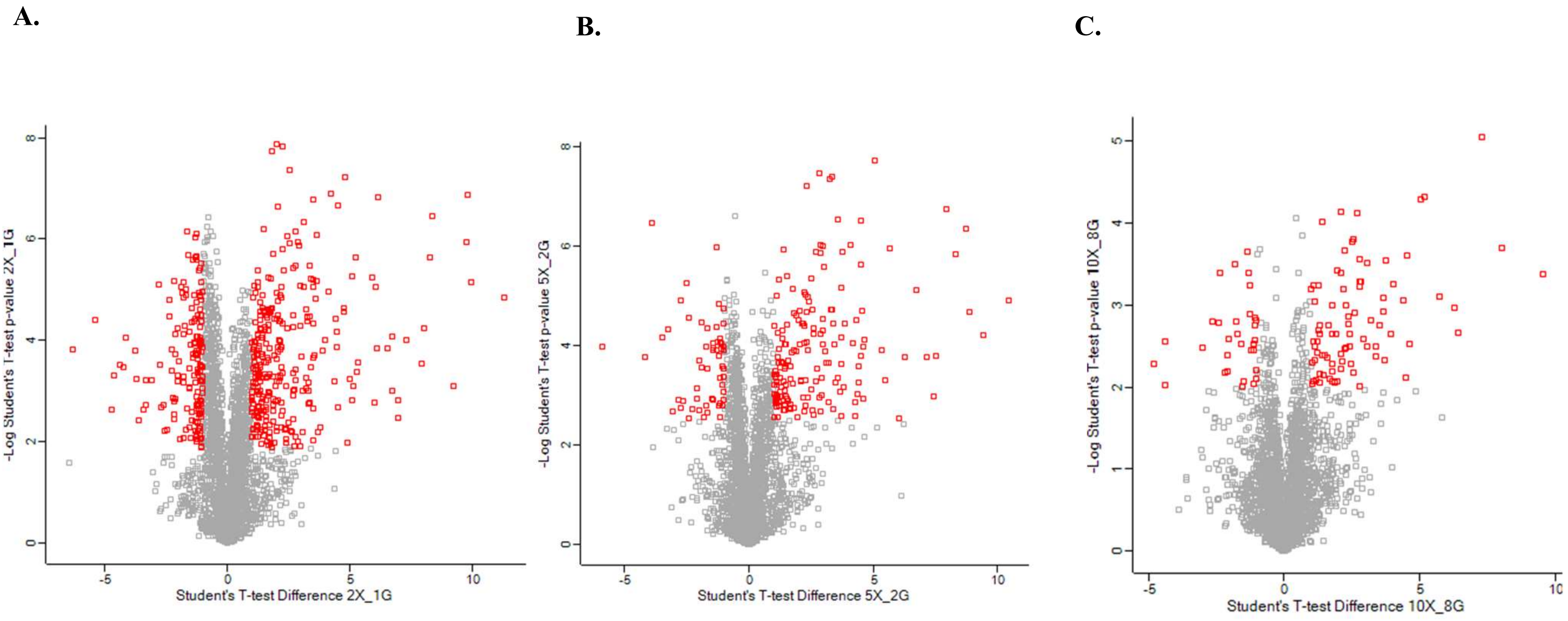

Fig. S3. Volcano plots of differentially expressed proteins in *R. toruloides* cultivated on either glucose or xylose during early exponential growth phase (A), late exponential growth phase (B), or lipid accumulation phase (C)

Fig. S4

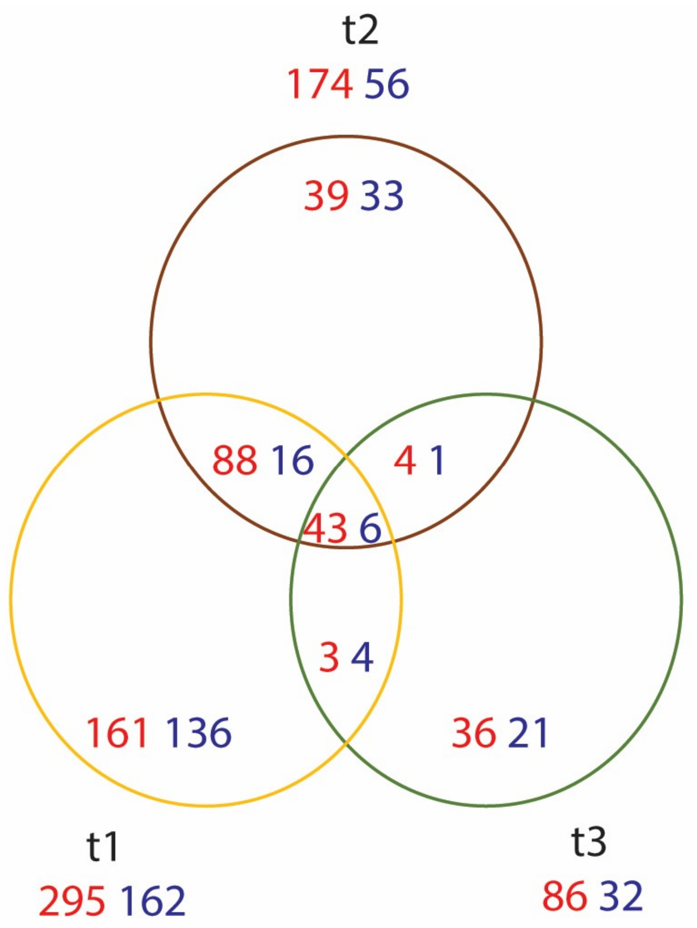

Fig. S4. Venn diagram of differentially expressed proteins in *R. toruloides* cultivated on either glucose or xylose during early exponential growth phase (t1, yellow circle), late exponential growth phase (t2, brown circle), or lipid accumulation phase (t3, green cycle). Number in red or blue colour indicates up or downregulated proteins, respectively, in cells grown on xylose as compared to cells grown on glucose.
